# Ingestion of Novaluron Elicits Transovarial Activity in *Stephanitis pyrioides* (Hemiptera: Tingidae)

**DOI:** 10.3390/insects11040216

**Published:** 2020-04-01

**Authors:** Shimat V. Joseph

**Affiliations:** Department of Entomology, University of Georgia, UGA Griffin Campus, 1109 Experiment Street, Griffin, GA 30223, USA; svjoseph@uga.edu; Tel.: +1-770-228-7312

**Keywords:** insect growth regulator, novaluron, azalea lace bug, azalea, spray

## Abstract

Azaleas (*Rhododendron* L. spp.) are widely grown ornamental plants in eastern and western regions of the USA. The azalea lace bug, *Stephanitis pyrioides* (Scott) (Hemiptera: Tingidae), is an important insect pest of azaleas. Adults and nymphs of *S. pyrioides* consume chlorophyll in azalea foliage, and severely affected plants appear bleached. Neonicotinoid insecticides are effective and widely used for *S. pyrioides* control; however, nursery growers and landscape professionals are concerned about nontarget effects on beneficial insects and demand neonicotinoid-free plants. There is clearly a need to develop reduced-risk control strategies for *S. pyrioides*. The insect growth regulator (IGR) novaluron elicits transovarial activity when adult *S. pyrioides* are exposed to it. However, it is not certain whether transovarial effects can be observed when *S. pyrioides* adults that colonize the abaxial leaf surface ingest novaluron residues deposited on the adaxial leaf surface. Experiments were conducted to assess transovarial activity upon exposure to various application rates of novaluron alone and novaluron with various adjuvants. The numbers of nymphs were significantly lower when the full rate of novaluron was applied on the adaxial surface of leaves compared to the number of nymphs on non-treated leaves. The densities of nymphs were not significantly different between the half and full rates of novaluron treatment. When novaluron with various adjuvants was applied to the adaxial surface of the leaves, the densities of nymphs were significantly lower under the novaluron treatments compared to the non-treated leaves, regardless of the type of adjuvant added. There was no significant difference between treatment with novaluron alone and the treatments of novaluron with adjuvants. These data show that transovarial activity was elicited in adults of *S. pyrioides* when novaluron was applied on the adaxial leaf surface.

## 1. Introduction

The azalea lace bug, *Stephanitis pyrioides* (Scott) (Hemiptera: Tingidae), can be a serious pest of azalea plants (*Rhododendron* L. spp; family Ericaceae) [1]. This pest is widespread in the landscape and represents a major problem for azalea producers in the eastern region of the USA. In addition, in the western USA, *S. pyrioides* is now established in Oregon and Washington, where it threatens *Rhododendron* L. spp. production in nurseries and is widespread in landscapes [2,3]. Both adults and all nymphal stages of *S. pyrioides* consume chlorophyll in foliage, which causes yellow speckles. As the pest population size increases, leaves become bleached or chlorotic in appearance with intense feeding activity [4,5,6]. Nursery plants infested with *S. pyrioides* are not marketed, and the infestation of established plants in landscapes reduces both aesthetics and property value [4,5]. Because damaged azalea leaves do not senesce or drop within a year, the impact of feeding injury is sustained for multiple years in the eastern USA.

*Stephanitis pyrioides* colonizes the abaxial leaf surface of azalea leaves [1]. Females implant eggs on both sides of the midrib [1]. *Stephanitis pyrioides* females often defecate a black, tar-like substance on newly implanted eggs, which appear as black spots on the abaxial leaf surface [6]. In the eastern USA, overwintering *S. pyrioides* eggs start hatching in March [7,8]. They undergo four overlapping generations in Georgia [8], whereas three generations are possible in Oregon and Washington [9]. For the management of *S. pyrioides*, it is advised to time the application as soon as first-generation nymph emergence has been observed. Thus, timely management can considerably restrict an increase in the *S. pyrioides* population during the growing season.

Typically, *S. pyrioides* are managed using neonicotinoids in nurseries as well as landscapes [10]. A single application of imidacloprid in granule or spray form in spring protects azalea plants from *S. pyrioides* for an entire year. However, the timing of neonicotinoid insecticide application often coincides with the active foraging of beneficial insects such as bees and wasps on blooming azaleas, especially in the spring. Because neonicotinoid use can pose a threat to pollinators and other beneficial arthropods, consumers demand neonicotinoid-free plants from nurseries and reduced use of neonicotinoid insecticides in landscapes. As an immediate response, nurseries and landscape professionals resort to the use of multiple spray applications of pyrethroid insecticides. Pyrethroid insecticides can also harm beneficial arthropods, which can cause secondary pest outbreaks [11]. To address the emerging pest management challenge faced by the nursery and landscape industry, novel and alternative options for *S. pyrioides* control are warranted.

The benzoylurea insecticide novaluron is registered for use in nurseries on several insect pests. It affects the biosynthesis of the insect cuticle [12,13] and is thus classified as a chitin biosynthesis inhibitor (Insecticide Resistance Action Committee, Group 15) [14]. As an insect growth regulator (IGR), novaluron is typically used to control immature stages of insect pests [15]. IGRs show minimal toxicity to mammals and beneficial insects, and they are therefore referred to as reduced-risk insecticides [15,16]. When novaluron was topically applied on adults of *S. pyrioides*, transovarial activity was observed, with the viability of the eggs being considerably reduced [17]. Similarly, when novaluron was applied to eggs and nymphs of *S. pyrioides*, ovicidal and nymphicidal activities, respectively, were observed [18]. Further studies are warranted to develop novaluron as an alternative tool and to incorporate it into integrated pest management programs for *S. pyrioides*.

For pest management, most insecticides are sprayed on plants, and insecticide residues are typically deposited on the adaxial leaf surface. Insect pests that are mobile on the leaf surface are easily exposed to insecticide residues, causing mortality by contact. However, insect pests that feed on abaxial leaf surfaces are exposed to insecticides via translaminar properties when insecticides penetrate the leaf surface and travel across the leaf blade. For example, the translaminar property of another IGR, pyriproxyfen, effectively controls the sweet potato whitefly, *Bemisia tabaci* (Gennadius), and the greenhouse whitefly, *Trialeurodes vaporariorum* (Westwood) [19]. *Stephanitis pyrioides* feeds on the abaxial leaf surface, and insecticide residues are typically deposited on the adaxial leaf surface. It is unclear whether *S. pyrioides* ingests novaluron residues while feeding, leading to the elicitation of transovarial activity. The objective of this study was to determine whether novaluron elicits transovarial activity against *S. pyrioides* through ingestion when novaluron residues are deposited on the adaxial leaf surface.

## 2. Materials and Methods

### 2.1. Plants and Insects

All the *S. pyrioides* adults used in the experiments were obtained from laboratory colonies maintained on live ‘George Tabor’ azalea plants in 3.7 L pots. The details of *S. pyrioides* rearing are described in [17,18]. At biweekly intervals, completely depleted plants were swapped with fresh non-infested plants to ensure uninterrupted food and water sources for *S. pyrioides* development. The plants used for rearing had no prior history of pesticide use. The plants with *S. pyrioides* were kept in cages under 40 W incandescent lamps that provided light and heat to the plants and *S. pyrioides*. The plants were maintained at ~55% relative humidity and temperatures of ~22 °C–39 °C (day:night), with a 16:8 h (light:dark) photoperiod. Under these laboratory conditions, the life cycle of *S. pyrioides* was completed within a month. The *S. pyrioides* populations in the rearing cages exhibited an ~1:1 sex ratio.

### 2.2. Insecticide and Adjuvants

The IGR used in the assays was novaluron (Pedestal^®^ [10% a.i.], OHP Inc., Bluffton, SC, USA). The rate of novaluron application was 58.1 g per ha. This rate was determined after referring to the Pedestal label for nursery use for *S. pyrioides*. The concentration of novaluron in the solution at the full rate of application was 155.4 ppm. The water volume was 373.9 L per ha, which was selected based on general practice in nursery production. Several adjuvants were used in various experiments. The adjuvants were as follows: (1) a nonionic surfactant, Dyne-Amic^®^ ([99% methyl esters of C16–C18 fatty acids, polyalkyleneoxide modified polydimethylsiloxane, and alkylphenol ethoxylate], Helena Agrichemicals, Collierville, TN, USA, (2) LI 700^®^ ([phosphatidylcholine, methylacetic acid, alkyl polyosyethylene and ether among 80 ingredients] Loveland Products, Inc, Greeley, CO, USA), (3) Liberate^®^ ([alcohol ethoxylates, lecithin, and methyl esters of fatty acids among 100 ingredients] Loveland Products, Inc., Greeley, CO, USA), and (4) Prescription Treatment^®^ brand Ultra-Pure Oil Horticultural Insecticide, Miticide and Fungicide ([petroleum oil, mixture of severely hydrotreated and hydrocracked base oil] St. Louis, MO, USA). The adjuvant was added to the novaluron treatments at 0.25% v/v.

### 2.3. Rates of Novaluron

The 3.7 L pots of ‘George Tabor’ azalea plants were used for this experiment. The treatments were 0×, 0.5×, and 1.0× of 58.1 g novaluron per ha. The treatments were arranged in a completely randomized block design with 10 replications in which an azalea branch terminal with 10–15 mature leaves served as the experimental unit. Novaluron solution was painted on the adaxial surface of the leaves using a fine-haired paint brush. The adjuvant Dyne-Amic was added at 0.25% v/v to all treatments, including the 0× treatment, in which tap water was used.

Sexually matured and mated *S. pyrioides* adults aged ~7 d were used in the experiment. For each replicate for all treatments included non-treated (0×), 10 adults were randomly collected from the rearing colony and caged on an azalea branch terminal for 4 d. A 14 × 11 cm sleeve mesh bag (length: width) was used as a cage. The end of the cage was secured to the azalea stem by pulling the cage’s strings. After 4 d of exposure, the adults were transferred from the branch terminals using hand-held aspirators and re-caged on new, non-treated branch terminals on a separate azalea plant. The previously exposed adults on the non-treated branch terminal were retained for 7 d. After 7 d of exposure, those adults were removed and were not used again in the experiment. The branch terminals that received novaluron treatment and non-treated branches were maintained for 14 more days so that the eggs oviposited on the foliage could hatch and develop. To prevent any *S. pyrioides* escape or reinfestation, these branches were caged again. The caged, potted plants were maintained in environmental control chambers at 28 °C and 75% relative humidity, under a 16:8 h (light:dark) photoperiod.

After 14 d for either 4 d novaluron-treated or 7 d non-treated branch terminals, the terminals were destructively removed for evaluation. The leaves were stripped from the branches and thoroughly examined for the presence of nymphs under a dissecting microscope. The nymphs observed on the leaf samples were separated into 1st-, 2nd-, 3rd-, 4th- and 5th-instar stages. The 1st instar is devoid of spines, pigmentation and wing pads, whereas the 2nd instar has spines. The 3rd instar has a pair of underdeveloped wing pad in addition to spines. The 4th instar has a pair of well-developed wing pads, whereas the 5th instar is large in size and has two pairs of wing pads. This experiment was repeated with five replications each time. The nymphs shed their translucent or white exoskeleton (exuviae) when they molt, and the number of exuviae per replicate was quantified. The number of defecation spots on the 4 d-exposed, novaluron-treated branch terminals was quantified to determine the feeding activity of *S. pyrioides* adults. The adults were introduced on May 24 and 31, 2019, for trials 1 and 2, respectively. The adults were transferred after 4 d of exposure on May 28 and June 4, 2019, for trials 1 and 2, respectively. The terminals exposed for 4 d were evaluated on June 11 and 18, 2019, for trials 1 and 2, respectively. Similarly, the terminals exposed for 7 d were evaluated on June 18 and July 2, 2019, for trials 1 and 2, respectively.

### 2.4. Novaluron Plus Adjuvant

The method used in this experiment was similar to that described in the previous section with a few exceptions. The experiments were conducted on ‘George Tabor’ azalea plants in 3.7 L pots. The treatments were as follows: (1) water, (2) novaluron only, (3) novaluron plus Dyne-Amic, (4) novaluron plus LI 700, (5) novaluron plus Liberate, and (6) novaluron plus Ultra Pure oil. The rate of novaluron application was 58.1 g per ha. In these treatments in which adjuvants were added, the rate applied was 0.25% v/v. The water control treatment received no adjuvant. The treatments were arranged in a completely randomized block design with 12 replications, in which an azalea branch terminal with 10–15 mature leaves served as the experimental unit. The treatments were painted on the adaxial surface of the leaves using a fine-haired paint brush.

As indicated in the previous section, 10 *S. pyrioides* adults were randomly collected and caged on a terminal branch for 4 d, and the adults were then transferred to a non-treated terminal branch for 7 d. After 7 d of exposure, the adults were removed and discarded. All the terminals exposed to *S. pyrioides* were caged again to avoid any *S. pyrioides* reinfestation or escape, and these conditions were maintained for 14 d before evaluation. The caged potted plants were maintained in environmental control chambers at 28 °C with 75% relative humidity and a 16:8 h (light:dark) photoperiod.

After the 14 d period, terminals that had been subjected to either 4 d or 7 d of exposure were destructively removed, and their leaves were thoroughly examined for *S. pyrioides* nymphs. The nymphs observed on the leaf samples were separated into 1st-, 2nd-, 3rd- 4th- and 5th-instar stages. The shed skin or exuviae were quantified. For the 4 d-exposed terminals, the number of defecation spots was quantified. This experiment was repeated four times with 3 replications each time. The adults were introduced on September 27, October 18, and December 2 and 16, 2019, for trials 1, 2, 3 and 4, respectively. The adults were transferred after 4 d of exposure on October 1 and 22 and December 6 and 20, 2019, for trials 1, 2, 3 and 4, respectively. The terminals exposed for 4 d were evaluated on October 15, November 5 and December 20, 2019, and January 3, 2020, for trials 1, 2, 3 and 4, respectively. Similarly, the terminals exposed for 7 d were evaluated on October 22, November 12 and December 27, 2019, and January 10, 2020 for trials 1, 2, 3 and 4, respectively.

### 2.5. Statistical Analysis

All statistical analyses of the data were performed in SAS [20]. The data from all the repeated trials for each experiment type were combined because the individual experiments were repeated and evaluated using exactly the same protocols and conditions. The nymphs were combined into groups of young instars (1st and 2nd instars) and old instars (3rd, 4th and 5th instars) before analysis. Hereafter, they are referred to as young and late instars. To determine the effects of novaluron rates, the data from young and old instars and all nymphs as well as the exuvium and defecation spot density data were square root transformed and then subjected to one-way ANOVA using a general linear model (PROC GLM) in which the novaluron rates were the treatments. Similarly, one-way ANOVA was performed on the data from young and late instars, total nymphs, exuviae and defecation spots from the novaluron plus adjuvant experiment after square root transformation using a general linear model (PROC GLM) in which the various novaluron plus adjuvant conditions were the treatments. For both experiments, the means were separated using Tukey’s HSD test for treatment comparisons. All the statistical comparisons were considered significant at α = 0.05.

## 3. Results

### 3.1. Rates of Novaluron

**Figure 1:**
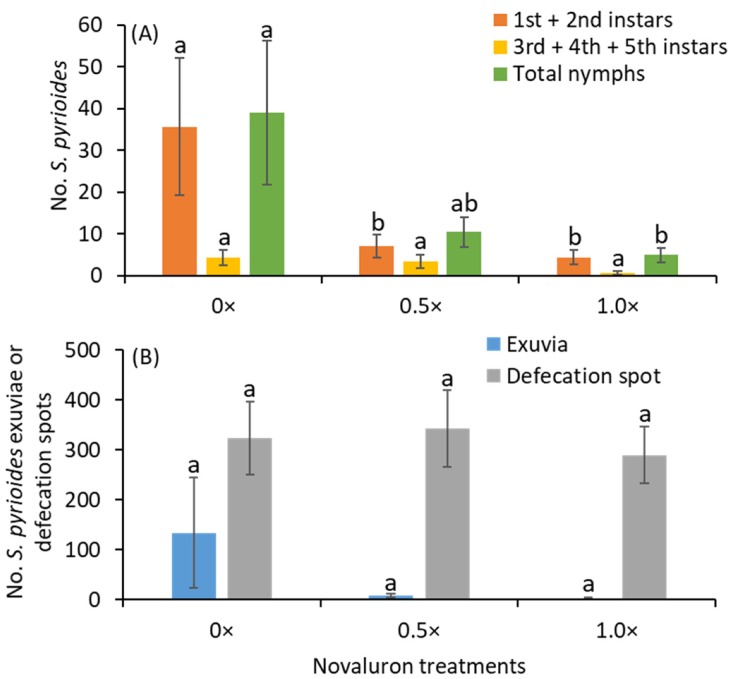
Mean (±SE) (**A**) numbers of various instars and total nymphs of *S. pyrioides* and (**B**) exuviae and defecation spots after adults fed for 4 d on the abaxial leaf surface of leaves treated with novaluron on the adaxial surface. Bars with the same fill color with the same letters are not significantly different (Tukey’s HSD test, *p* = 0.05).

After 4 d of exposure to novaluron-treated leaves, the numbers of young instars (1st + 2nd instars) were significantly lower in the 0.5× or 1.0× treatments than in the 0× treatment (*F*_2, 11_ = 6.2; *p* = 0.016; Figure 1A). There were no significant differences between the 0×, 0.5× or 1.0× treatments for old instars (3rd + 4th + 5th instars) after 4 d (*F*_2, 11_ = 1.6; *p* = 0.247). The total numbers of nymphs were significantly lower in the 1.0× treatment than in the 0× treatment (*F*_2, 11_ = 5.4; *p* = 0.023). However, the densities of exuviae (*F*_2, 11_ = 1.6; *p* = 0.239) and defecation spots (*F*_2, 11_ = 0.4; *p* = 0.682) were not significantly different between the 0×, 0.5× and 1.0× treatments (Figure 1B).

**Figure 2:**
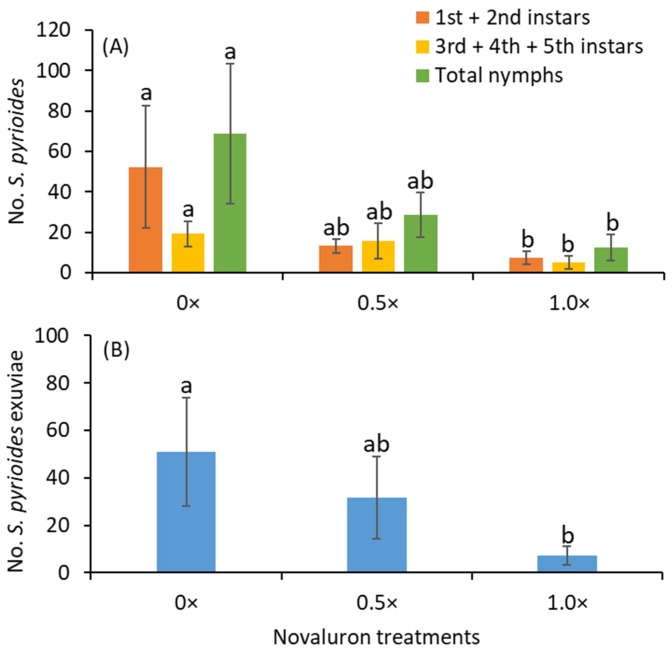
Mean (±SE) (**A**) numbers of various instars of *S. pyrioides* and (**B**) exuviae when adults that ingested novaluron were allowed to oviposit for 7 d on insecticide-free plants. Bars with the same fill color and the same letters are not significantly different (Tukey’s HSD test, *p* = 0.05).

After 4 d of exposure, the adults were transferred to non-treated foliage for 7 d. After 7 d of exposure to non-novaluron-treated leaves, the numbers of young instars (*F*_2, 16_ = 5.0; *p* = 0.020), old instars (*F*_2, 16_ = 6.6; *p* = 0.008) and total nymphs (*F*_2, 16_ = 5.8; *p* = 0.013) were significantly lower in the 1.0× treatment than in the 0× treatment (Figure 2A). There were no significant differences between the 0× and 0.5× or 0.5× and 1.0× treatments for the numbers of young and old instars or total nymphs. The exuviae densities were significantly lower in the 1.0× treatment than in the 0× treatment (*F*_2, 16_ = 5.3; *p* = 0.017; Figure 2B).

### 3.2. Novaluron Plus Adjuvant

**Figure 3:**
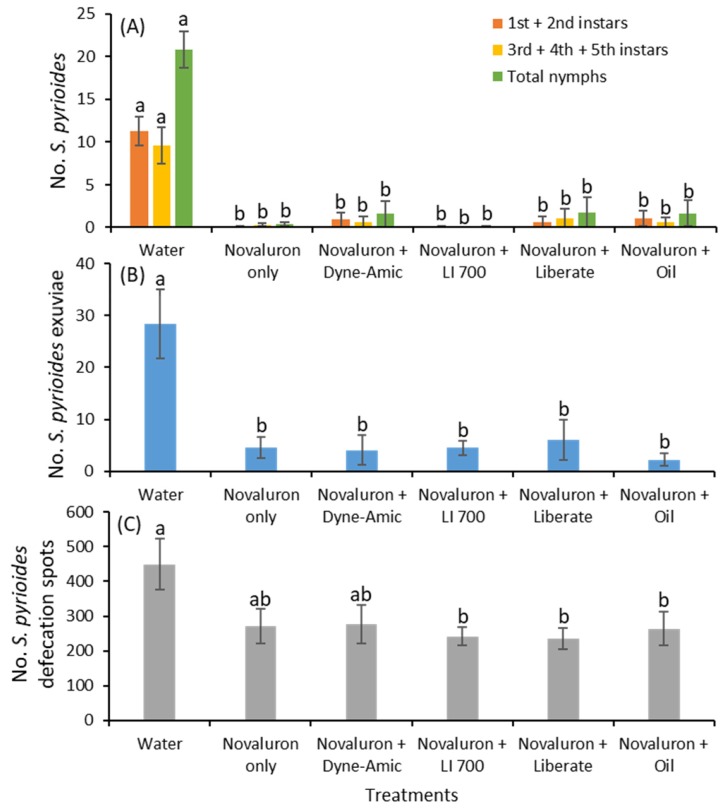
Mean (±SE) (**A**) numbers of various instars and total nymphs of *S. pyrioides*, (**B**) exuviae and (**C**) defecation spots after adults fed for 4 d on the abaxial leaf surface of leaves treated with novaluron and novaluron + various adjuvants on the adaxial surface. Bars with the same fill color and the same letters are not significantly different (Tukey’s HSD test, *p* = 0.05).

After 4 d of exposure to residues of novaluron alone or novaluron plus adjuvants, the numbers of young (*F*_5, 55_ = 29.9; *p* < 0.001) and old instars (*F*_5, 55_ = 13.7; *p* < 0.001) as well as total nymphs (*F*_5, 55_ = 32.3; *p* < 0.001) were significantly lower for novaluron only, novaluron plus Dyne-Amic, novaluron plus LI 700, novaluron plus Liberate, and novaluron plus oil compared to the water control treatment (Figure 3A). There were no significant differences for the young (*F*_5, 55_ = 29.9; *p* < 0.001), old instars, and total nymphs between the novaluron alone and novaluron plus various adjuvant treatments. Similarly, the densities of exuviae were significantly lower for all novaluron plus various adjuvants and novaluron alone treatments than for the water control treatment (*F*_5, 55_ = 9.9; *p* < 0.001; Figure 3B). The numbers of defecation spots were significantly greater under the water control treatment than in the novaluron plus LI 700, novaluron plus Liberate and novaluron plus oil treatments (*F*_5, 55_ = 2.9; *p* = 0.019; Figure 3C).

**Figure 4:**
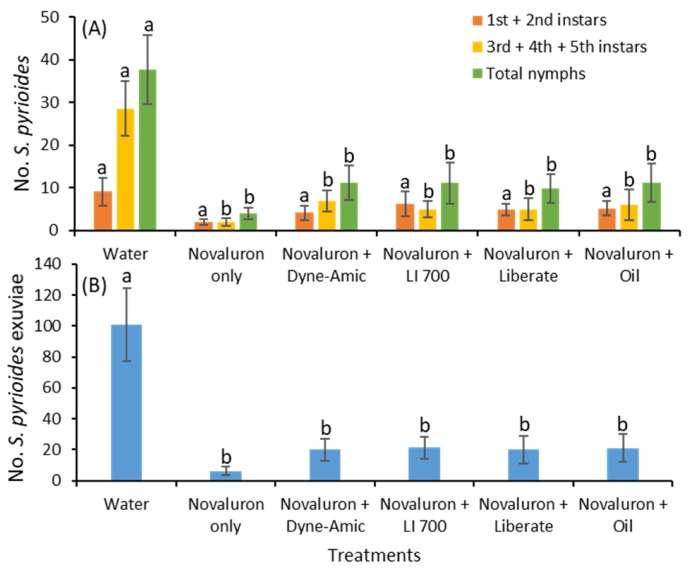
Mean (±SE) (**A**) numbers of various instars of *S. pyrioides* and (**B**) exuviae when adults that ingested novaluron and novaluron + various adjuvants were allowed to oviposit for 7 d on insecticide-free plants. Bars with the same fill color with the same letters are not significantly different (Tukey’s HSD test, *p* = 0.05).

After 4 d of exposure, the adults were transferred to non-treated foliage for 7 d. After 7 d of exposure to non-novaluron-treated leaves, the results were not different from those after 4 d of exposure. The numbers of young instars were not significantly different between the novaluron alone or novaluron plus adjuvant treatments and the water control treatment (*F*_5, 55_ = 2.0; *p* = 0.087; Figure 4A). The densities of old instars (*F*_5, 55_ = 9.8; *p* < 0.001) and total nymphs (*F*_5, 55_ = 8.5; *p* < 0.001) were significantly lower for all the novaluron treatments compared to the water control treatment. There were no significant differences for the young and old instars or the total nymphs between the novaluron alone and novaluron plus various adjuvant treatments. Similarly, the densities of exuviae were significantly lower under all novaluron plus various adjuvant and novaluron alone treatments than in the water control treatment (*F*_5, 55_ = 11.5; *p* < 0.001; Figure 4B).

## 4. Discussion

The results showed that transovarial activity was elicited in the adult *S. pyrioides* that colonized at the abaxial leaf surface when novaluron was applied on the adaxial leaf surface. This is important new information that has practical implications for operations such as landscape maintenance businesses and nurseries in which insecticides are routinely sprayed on plants and their residues are normally deposited on the adaxial or upper leaf surface. For a pest such as *S. pyrioides* that colonizes and feeds from the abaxial leaf surface, it is critical that the insecticide has translaminar or systemic properties to deliver the lethal dose. Novaluron has demonstrated translaminar properties and exerts activity against insect pests through ingestion as well as contact [16]. A previous study showed that among novaluron, buprofezin, pyriproxyfen and azadirachtin, novaluron was by far the most effective IGR, with transovarial effects on adult *S. pyrioides* [17]. In addition, another study showed that nymphs of *S. pyrioides* were susceptible to novaluron when the residues were deposited on the adaxial side of the leaves [18]. This suggests that novaluron can be effective in reducing field populations of *S. pyrioides* by affecting egg hatching through transovarial activity as well as disrupting nymphal development through abnormal ecdysis. The early-season foliar spraying of novaluron can target the first generation of emerging young instars or adults and effectively suppress the population increase later in the season.

When the half rate of novaluron was compared with the full rate, the numbers of *S. pyrioides* nymphs that emerged were found to be similar between these two rates. This suggests that adults can ingest a lower dose of novaluron from the abaxial leaf surface and still show transovarial effects. The presence of defecation spots after 4 d of exposure regardless of the novaluron rates applied indicates that the adults of *S. pyrioides* actively fed on the azalea leaves and ingested residues of novaluron applied on the adaxial leaf surface. The numbers of defection spots on the abaxial leaf surface are associated with increased feeding activity and injury [6]. A previous study showed that even a quarter rate of novaluron was as effective as the full rate in eliciting transovarial effects when novaluron was directly applied to the adults of *S. pyrioides* [17]. Based on the current and previous studies, it is clear that *S. pyrioides* is very sensitive to novaluron, even at lower doses, implying that novaluron is likely to be effective in suppressing the *S. pyrioides* population under field conditions.

The data showed that adjuvants did not improve the transovarial effects in *S. pyrioides* compared with novaluron. The leaves treated with novaluron alone and novaluron with any adjuvant exerted similar effects on nymphal densities. In contrast, a previous study showed that adding an adjuvant improved the ovicidal activity of novaluron in *S. pyrioides* [18]. This suggests that adding an adjuvant to novaluron may present value in suppressing the *S. pyrioides* population in the long run. The evidence showed that adult *S. pyrioides* actively fed on foliage treated with novaluron alone or novaluron plus any adjuvant or the water control similarly, as the tar-like substance was present on all the treated leaves. The lack of improvement in transovarial activity in adults after adding the adjuvant is related to the translaminar movement of novaluron. Adults and nymphs of *S. pyrioides* insert their stylets through stomatal openings to consume chlorophyll in the parenchyma cells located in the epidermal layer of the leaves [1]. This means that the deposited novaluron residues are not required to travel through several cell layers to be accessible to adults and nymphs of *S. pyrioides*. Although the length of the stylets may be shorter in the first and second instars of *S. pyrioides* than in adults, the effects of novaluron were evident even in young nymphs [18], which suggests that novaluron without any adjuvant can cross the leaf blade and intoxicate young nymphs through ingestion. A previous study also showed that young instars directly exposed to novaluron residues on the leaf surface did not exhibit mortality [18].

## 5. Conclusions

The data showed that when the adaxial leaf surface was treated with novaluron, reduced numbers of nymphs and their exuviae were observed compared to the water control, suggesting that *S. pyrioides* elicits transovarial activity upon exposure through ingestion. This result indicates that novaluron can be applied as a traditional foliar spray and can suppress *S. pyrioides* populations developing on abaxial leaf surfaces. The half rate of novaluron was as effective as the full dose in eliciting transovarial effects. The addition of an adjuvant to novaluron did not provide any added benefits toward improving the transovarial activity of *S. pyrioides*. These results suggest that the IGR novaluron offers an alternative option to use neonicotinoid insecticides for *S. pyrioides* management. Additionally, pyrethroid insecticides can be replaced by the adoption of IGRs, especially novaluron, for the integrated pest management of *S. pyrioides*.
